# Supplementary material for: Anti-Metalloproteases: Production and Characterization of Polyclonal IgG Anti-F2 Fraction Antibodies Purified from the Venom of the Snake Bitis arietans
Source: Toxins (Basel). 2023 Apr 1;15(4):264. doi: 10.3390/toxins15040264 (PMC10145261; doi:10.3390/toxins15040264)
Supplement: Supplementary file 1 [file toxins-15-00264-s001.zip › toxins-2283922-supplementary.pdf]

Supplementary material

Identification of the peptides corresponding to the bands of the venoms of snakes by mass spectrometry

Selected bands from the polyacrylamide gel (Bg, Bn, Br, F2.1 and F2.2) obtained in *2.7* were submitted to in gel digestion and identified by mass spectrometry. The data obtained were submitted to bioinformatic analysis using BLASTP 2.13.0 [31], UniProtKB/SwissProt, and PEAKS DB, and submitted to a search in the "Serpentes" database (taxid: 8570). Supplementary table S1 lists the most abundant proteins found in each analyzed sample. The low presence of peptides such as SVMPs, disintegrin, and snaclecs explains the poor recognition of bands from *B. gabonica*, *B. nasicornis,* and *B. rhinoceros* by anti-F2 fraction antibodies. In the F2 fraction, it was possible to identify peptides from the SVMPs and Snacles families, in addition to peptides that match the composition of the venom.

Supplementary table S1. Identification of proteins from electrophoresis gel bands of *Bitis* venoms and the F2 fraction.

Fraction			
	**Sequence[a]**	**peptide**	**class[b]**
**Bg**	RLDEAEQLALKG	Myosin	ATPase
**Bg**	RGYSFVTTAERE	Actin	Globular proteins that form microfilaments
**Bg**	KLTPGSQC	Bitistatin	SVMP/disintegrin
**Bg**	KIGGIGTVPVGRV	Elongation factor	tRNA
**Bg**	KLRVDPVNFKF	Hemoglobin	Metalloprotein
**Bg**	KAQYEDIAQKS	Keratin	Fibrous protein
**Bg**	RAGLQFPVGRI	Histone	Histones
**Bg**	KSTTDLPRF	L-amino acid oxidase	LAAO
**Bn**	KLHSWVEC	SVMP P-III	SVMP
**Bn**	KLAIVADHRMYTKY	Disintegrin-like metalloprotease	SVMP/disintegrin
**Bn**	KTTDNQWLRW	Snaclec CTL	Snaclec
**Bn**	KLLVVYPWTQRF	Beta hemoglobin	metalloprotein

**Bn**	RDVNRASEL	L-amino acid oxidase	LAAO
**Br**	KNDLQLQVQAEADGLADAEERC	Myosin	ATPase
**Br**	KIPCAPQDVKC	SVMP P-III	SVMP
**Br**	KTFIVADKRM	Disintegrin-like metalloprotease	SVMP/disintegrin
**Br**	KLTPGSQC	Bitistatin – (SVMP)	SVMP/disintegrin
**Br**	KIGGIGTVPVGRV	Elongation factor	tRNA
**Br**	KTTDNQWLRW	Keratin	Fibrous protein
**F2.1**	KSYELPDGQVITIGNERF	Bothropasin/Jararagin – (SVMP)	SVMP
**F2.1**	RDLTDYLM	Snaclec PAL	Snaclec
**F2.1**	KSYELPDGQVITIGNERF	Bothropasin – (SVMP)	SVMP
**F2.1**	RHQGVMVGMGQKD	Atrolysin A – (SVMP)	SVMP
**F2.1**	KIWHHTFYNELRV	VEGF-Fs barietin	VEGF-Fs
**F2.1**	KSYELPDGQVITIGNERF	Bothropasin – (SVMP)	SVMP
**F2.2**	KTWEDAEKF	Bitiscetin	Snaclec
**F2.2**	RVAPEEHPTLLTEAPLNPKA	Alpha actin	Globular proteins that form microfilaments
**F2.2**	KSYELPDGQVITIGNERF	Bothropasin – (SVMP)	SVMP
**F2.2**	KEITALAPSTMKI	BA-5A – (SVMP-PII)	SVMP
**F2.2**	RGYSFVTTAERE	Hyaluronidase (*Bitis arietans*)	Hyaluronidase
**F2.2**	KIWHHTFYNELRV	VEGF-Fs barietin	VEGF-Fs
**F2.2**	RVVESMQSTLDAEIRS	Snaclec	Snaclec
**F2.2**	RIQLELNQVKS	Bistiscetin	Snaclec
**F2.2**	RSLSTELFKM	PLA2	PLA
**F2.2**	RIQLVEEELDRA	Batroxycidin	antimicrobial peptide
**F2.2**	KAQYEDIAQKS	SVSP	SVSP

The peptide sequences of fragments found in the bands of *B. gabonica*, *B. nasicornis*, *B. rhinoceros* venoms, and the F2 fraction were analyzed by mass spectrometry (LC-MS/MS), using BLASTP 2.13.0 [31], UniProtKB/SwissProt and PEAKS DB, and searched the "Serpentes" database (taxid:8570). [a] The According to PEAKS DB. [b] Classes of toxins; ATPase, adenosine triphosphatase; SVSP, snake venom serine protease; SVMP, snake venom metalloproteinase; tRNA, transporter ribonucleic acid; LAAO, L-amino acid oxidation; VEGF-Fs, snake venom vascular endothelial growth factor toxin.
